# Spatial Health Predictors for Depressive Disorder in Manhattan: A 2020 Analysis

**DOI:** 10.7759/cureus.41607

**Published:** 2023-07-09

**Authors:** Vincent Giordano, Tara Rigatti, Asad Shaikh

**Affiliations:** 1 Geography and Cartography, Kent State University, Kent, USA; 2 Trauma Social Work, Richmond University Medical Center, Staten Island, USA; 3 Psychiatry, Richmond University Medical Center, Staten Island, USA

**Keywords:** geo-temporal analysis, mental wellbeing, urban environment, urban and rural community, public health care

## Abstract

Background

Urban cores often present extreme disparities in the distribution of wealth and income. They also vary in health outcomes, especially regarding mental welfare. Dense urban blocks agglomerate many residents of various backgrounds, and extreme differences in income, commerce, and health may lead to variations in depressive disorder outcomes. More research is needed on public health characteristics that may affect depression in dense urban centers.

Methods

Data on 2020 public health characteristics for Manhattan Island was collected using the Centers for Disease Control’s (CDC's) PLACES project. All Manhattan census tracts were used as the spatial observations, resulting in $N=281$ observations. A cross-sectional generalized linear regression (GLR) was used to fit a geographically weighted spatial regression (GWR), with tract depression rates as the endogenous variable. Data on the following eight exogenous parameters were incorporated: the percentage without health insurance, the percentage of those who binge drink, the percentage who receive an annual doctor’s checkup, the percentage of those who are physically inactive, the percentage of those who experience frequent mental distress, the percentage of those who receive less than 7 hours of sleep each night, the percentage of those who report regular smoking, and the percentage of those who are obese. A Getis-Ord Gi* model was built to locate hot and cold spot clusters for depression incidence and an Anselin Local Moran's I spatial autocorrelation analysis was undertaken to determine neighborhood relationships between tracts.

Results

Depression hot spot clusters at the 90%-99% confidence interval (CI) were identified in Upper Manhattan and Lower Manhattan using the Getis-Ord Gi* statistic and spatial autocorrelation. Cold spot clusters at the 90%-99% CI were in central Manhattan and the southern edge of Manhattan Island. For the GLR-GWR model, only the lack of health insurance and mental distress variables were significant at the 95% CI, with an adjusted R­^2^ of 0.56. Noticeable inversions were observed in the spatial distribution of the exogenous coefficients across Manhattan, with a higher lack of insurance coefficients observed in Upper Manhattan and higher frequent mental distress coefficients in Lower Manhattan.

Conclusion

The level of depression incidence does spatially track with predictive health and economic parameters across Manhattan Island. Additional research is encouraged on urban policies that may reduce the mental distress burden on Manhattan residents, as well as investigations of the spatial inversion observed in this study between the exogenous parameters.

## Introduction

Residents of urban cores, on average, enjoy a higher standard of living and greater access to opportunity than their rural counterparts. These urban regions agglomerate high concentrations of wealth, institutions, and health centers in dense blocks, allowing for continuous spillovers of these positive attributes for residents. However, urbanization also leads to extreme variations in the distribution of wealth and resources with high levels of segregation by block. One area of disparity is in healthcare outcomes. While urban residents are more well-connected to spatial concentrations of medical providers, the variations in socioeconomic characteristics deprive many of critical health services such as periodic primary care checkups, cancer screenings, and radiology imaging. Regular access to physical fitness, stable rapid eye movement (REM) sleep schedules, and health habits also vary spatially. Nevertheless, urban populations generally face lower rates of clinical depression and medically diagnosed depression symptoms. One study estimated that a single unit increase in the size and scale of an urban center led to a reduction in depression rates of 0.132 percentage points [1].

Yet, a growing number of cities have seen rising rates of diagnosed depressive disorder and clinical depression symptoms. One such city is New York, with the island of Manhattan exhibiting extreme variations. Upper Manhattan’s East Harlem neighborhood had the highest rate of current depression prevalence in the city at 21.4% while neighboring Central Harlem and Morningside Heights had one of the lowest citywide rates of 5.3% [2]. Such sharply disjointed statistics for completely adjacent city neighborhoods may be explained by the extremely segregated and gentrified nature of urban blocks. Additionally, significant concentrations of clinically depressed residents of New York City can be found in the majority non-White neighborhoods and areas with high levels of poverty, substance abuse, and physical inactivity. Compounding this problem, symptomatic depression, or depression with clinically recorded medical symptoms, led to significant daily delays in regular activities for residents of New York City [3].

Research indicates some modest negative relationship between physical exercise and depression. Physical activity has been shown to lower incident depression while also reducing general symptoms of major depressive disorder (MDD) [4]. In a similar vein, supervised exercise regimens administered by clinicians led to more consistent physical activity patterns and thus reduced depressive symptoms [5]. Furthermore, longitudinal analyses of the vast literature on exercise and depression concluded that exercise is not widely used as a form of therapy due to prevalent misconceptions such as age-related fragility and neurochemical reactions [6].

Primary care providers (PCPs) are the first clinicians to encounter depression symptoms during routine visits but are often the least equipped to treat them. Nevertheless, regular visits to PCPs have been correlated with earlier detection of anxiety and depression among older patients. Most of these patients prefer to treat depression and other anxiety-related disorders via their PCPs and family practitioners [7]. In addition, United States health officials recommend improved and targeted screening assessments to identify depression symptoms during PCP visits and other routine checkups to better assist PCPs in the initial identification and management phases [8]. 

Depression also shared a dependent relationship with nicotine addiction and cigarette usage. Boden et al. found a causal impact of nicotine dependence on depression symptoms; specifically, those aged 17-24 with severe nicotine dependence were 2.13 times as likely to report clinical symptoms of depression than their non-dependent peers [9]. Similarly, excessive routine alcohol consumption, or “binge drinking,” led to higher depression incidence, with both nicotine and alcohol dependence causing unpredictable mood swings, incapacitated emotional regulation, and prolonged feelings of anxiety [10]. 

Regarding other physical comorbidities, obesity, and depression appear to have some positive correlation, with obese patients having a 55% higher chance of being diagnosed with depressive disorders. The relationship is bidirectional, with depressed patients being at a 58% increased risk for obesity. While exact causes are debated by clinicians, it is believed that inflammation responses arising from long-term obesity cause neurochemical imbalances which affect moods and feelings of anxiety. Furthermore, obesity may lead to negative perceptions of oneself and fear of societal prejudice, significantly affecting a person’s feeling of self-worth [11]. Another comorbidity that affects depression is prolonged sleep deprivation. For adolescents aged 11-17, Roberts and Duong found that sleep periods of 6 hours or less put patients at 3.79 times increased risk for MDD and 1.42 times increased risk for general depression symptoms [12]. 

Beyond physical and mental health characteristics, economic circumstances have been shown to influence population and community mental health. Early detection of depressive disorder symptoms may prevent long-term MDD and severe anxiety disorders. However, financial constraints may prevent patients from routine PCP visits which would prove invaluable in said early detection. Twenty-three percent of U.S. citizens reporting symptoms of depression avoided treatment due to an inability to pay, with a significant portion lacking health insurance. Fity-five percent of those aged 18-26 did not receive treatment due to cost, coverage transitions, and lack of resources to locate providers [13]. Black respondents made up a disproportionate share of this pool, at 53% of the total number avoiding treatment.

While these attributes may indeed predict depressive disorders in many different settings, the effects may be felt most acutely in cities despite their overall better outcomes than less developed areas. Non-White residents tend to make up significant shares of the gentrified and decaying areas of urban cores. These residents often face significant hurdles in accessing mental health treatment, addiction counseling, and drug rehabilitation, with major spatial “cold spots” of these vital amenities in their immediate vicinities. Spatial trends in economic and residential segregation may also produce a patchwork of inverted adjacent depression rates by city blocks not found in the countryside. This study, therefore, seeks to establish spatial connections between the overall density of a major urban region and its relationship between health characteristics and clinical depression. It specifically investigates the spatial statistical relationships urban neighborhood health disparities have with clinical depression in Manhattan, a region with a population density of 74,781.6 people per square mile [14]. A geographically weighted regression (GWR) was estimated using exogenous parameters to reasonably predict depression rates and their spatial attributes. To examine the spatial distribution of depression rates, both a Getis-Ord Gi* statistic and an Anselin Local Moran’s I spatial autocorrelation were calculated by census tract.

## Materials and methods

**Figure 1:**
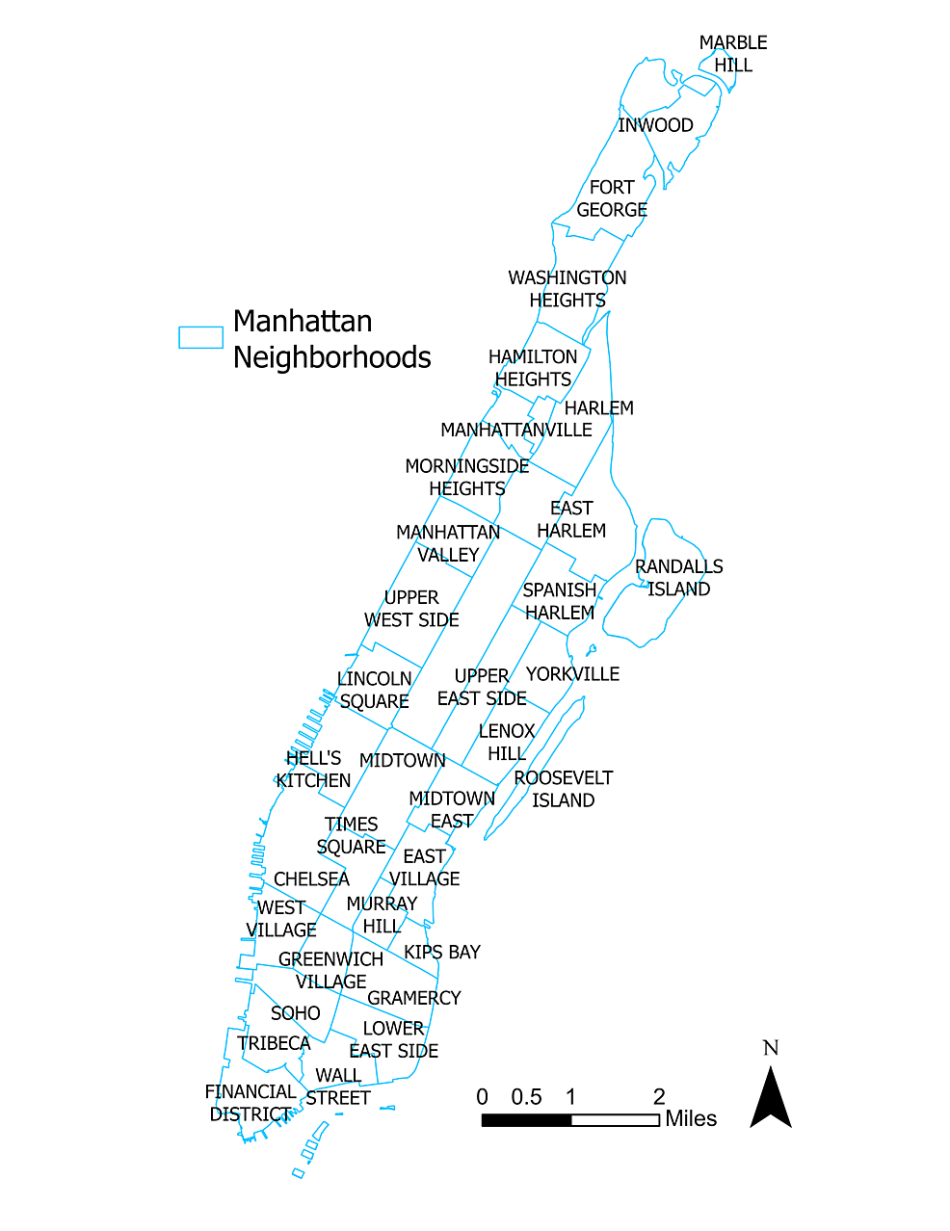
Manhattan neighborhood boundaries.

**Figure 2:**
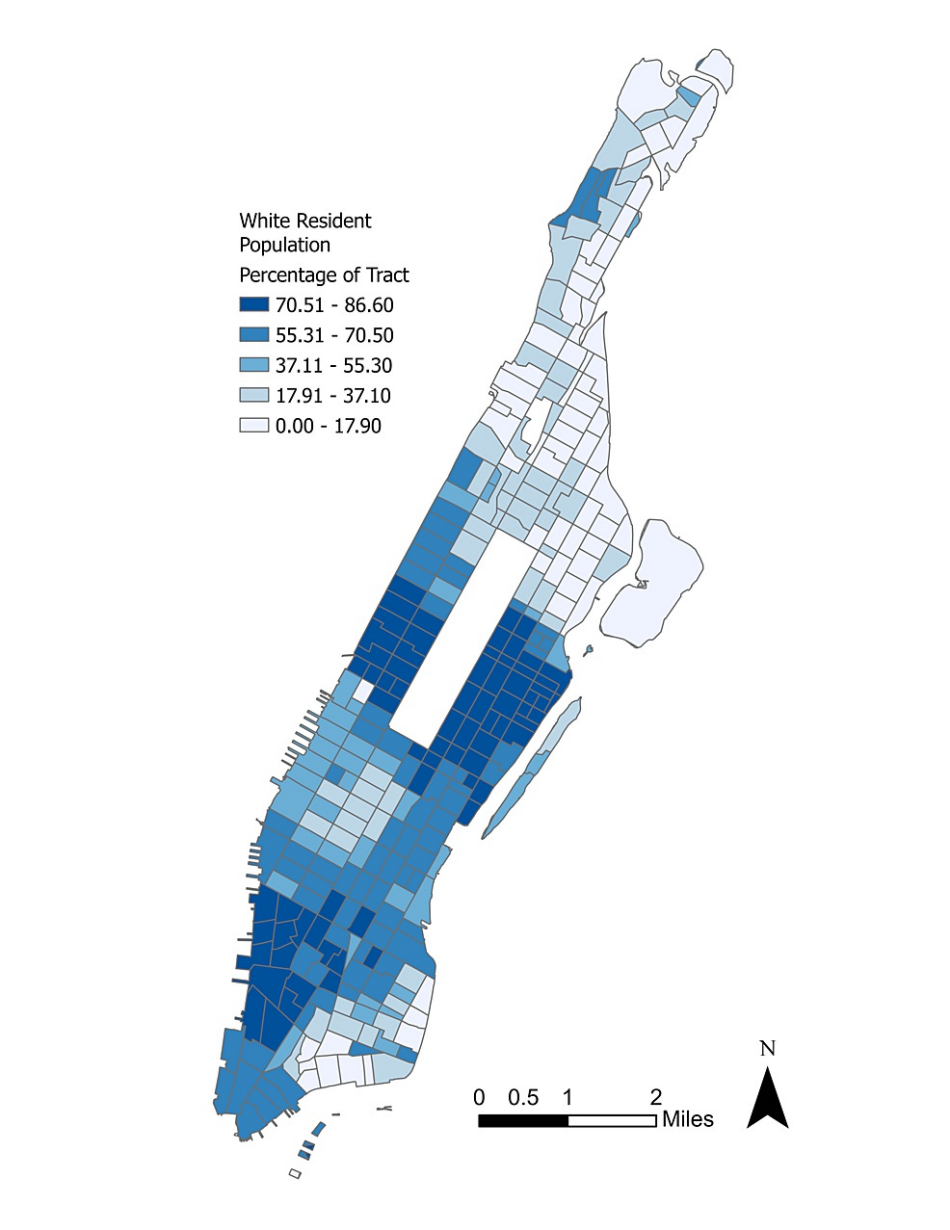
White resident population by Manhattan 2020 census tract.

The study area was selected as the Borough of Manhattan in the City of New York, which constitutes an area of sufficient racial and ethnic diversity, robust commerce, and heavy urban population density. Figure 1 details the distribution of neighborhoods and their boundaries and Figure 2 illustrates the White resident population, which will both be referred to when discussing the spatial distribution of the results.

Neighborhoods in Upper Manhattan, including Inwood, Washington Heights, and Harlem tend to be majority non-White, have lower household incomes, and face significant barriers to mental health treatment. In contrast, Manhattan’s wealthiest neighborhoods, such as the Upper East Side, the Upper West Side, and SoHo, concentrate in Midtown and Lower Manhattan and tend to be majority White, have higher household incomes, and enjoy greater access to mental care facilities and treatment centers.

To investigate the spatial distribution of mental health and treatment in Manhattan, data was imported into ArcGIS Pro on all Manhattan census tracts using the Centers for Disease Control and Prevention’s (CDC) PLACES Project 2020 datasets, which compiled data from 2017-2018 [15]. This resulted in total sample observations of $N=281$. Poor mental health outcomes were measured using clinical depression statistics, or the percentage of tract residents ≥ 18 years of age who were diagnosed with depressive disorder under the Diagnostic and Statistical Manual of Mental Disorders, Fifth Edition (DSM-5).

This study makes use of three types of spatial distribution analysis of depression. The first is the Getis-Ord Gi* statistic, which measures the clustering of existing concentrations of high or low point values at $0.01\leq \alpha \leq 0.10$ [16]. The second is Anselin Local Moran’s I, a more powerful form of hot spot analysis that also locates outlier values relative to existing clusters [17]. The third form of analysis is a GWR, which performs separate regression analyses for each spatial feature being studied (i.e., census tract) [18]. GWR helps rectify the paradox between the expected independence of observations and the geographic rule of spatial relationships between neighboring features [19]. For the GWR, data on eight predictive health variables were imported into ArcGIS Pro as described in Table 1.

**Table 1:** Regression predictive parameters for depressive disorder rates. Words in parentheses are the shorthand identifiers that will be referred to in the later regression model. PCP: Primary care provider. Kg/m^2^: Kilograms per square meter.

Variable	Description
Lack of health insurance (insurance)	Tract residents ≥ 18 years of age reporting no health insurance enrollment (%)
Binge drinking (drinking)	Tract residents ≥ 18 years of age reporting 4-5 drinks at a single sitting in the last 30 days (%)
Routine checkups (checkups)	Tract residents ≥ 18 years of age reporting a routine PCP visit in the previous year (%)
Smoking	Tract residents ≥ 18 years of age reporting daily or regular smoking of cigarettes (%)
Physical inactivity (inactivity)	Tract residents ≥ 18 years of age reporting no non-work physical exercise in the past month (%)
Frequent mental distress (distress)	Tract residents ≥ 18 years of age reporting poor mental health 14 days out of the previous 30 days (%)
Obesity	Tract residents ≥ 18 years of age reporting a body mass index (BMI) ≥ 30.0 kg/m^2^ (%)
Sleep deprivation (deprivation)	Tract residents ≥ 18 years of age reporting <7 hours of sleep regularly per night (%)

## Results

Getis-Ord Gi* model

**Figure 3:**
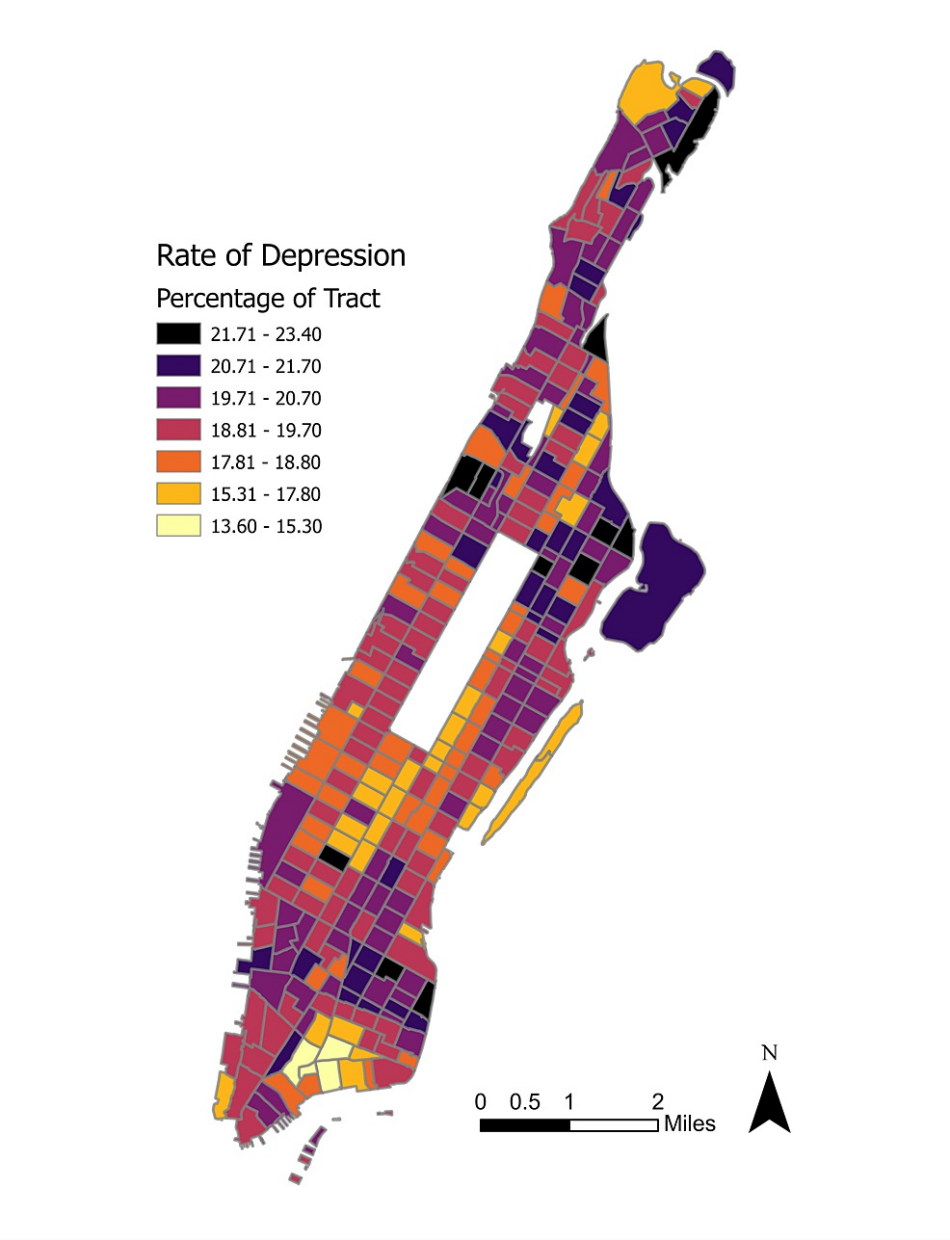
Rate of depression by Manhattan census tract.

**Figure 4:**
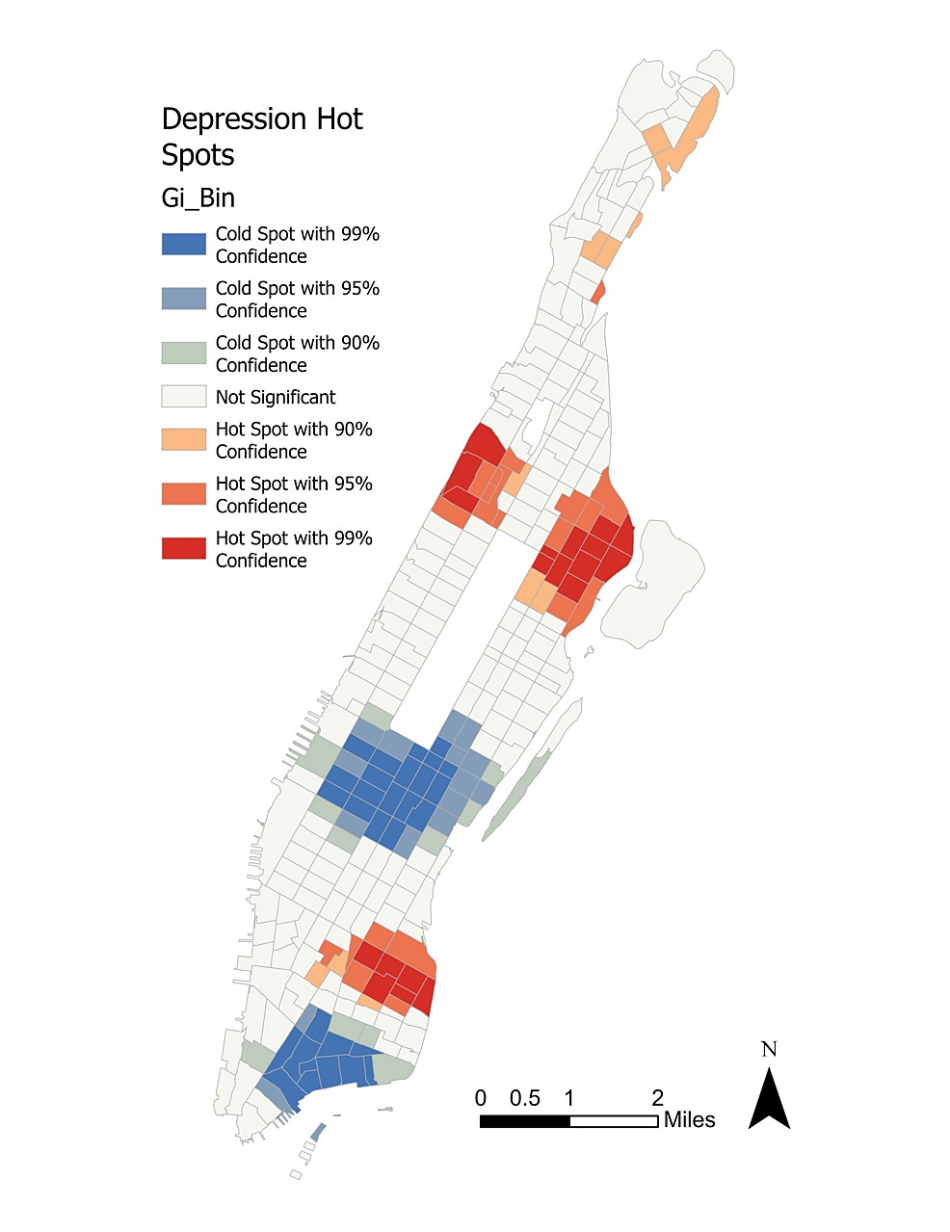
Getis-Ord Gi* hot spot results. Gi_Bin: The actual recorded hot or cold spot statistic, reported with a significance level between 0.01 and 0.10.

First, a Getis-Ord Gi* statistical model was run on depression incidence rates by census tract. Figure 3 shows the overall distribution of depressive disorder rates while Figure 4 shows the results of the initial cluster analysis.

Significant hot spot clusters at $0.01\leq \alpha \leq 0.05$ were observed in the neighborhoods of Spanish Harlem, East Harlem, Gramercy, and Morningside Heights. Less pronounced hot spots at $\alpha = 0.10$ were seen in Fort George and Inwood. Significant cold spots concentrated in much of Midtown, eastern Hell’s Kitchen, and Wall Street. A cold spot at $\alpha = 0.10$ is visible in southern Roosevelt Island. Upon first inspection, the distribution of clusters appears to modestly track with the neighborhood demographics as seen in Figure 2, with Upper Manhattan’s non-White majority neighborhoods experiencing the largest share of the correlation.

Local Moran’s I spatial autocorrelation analysis

**Figure 5:**
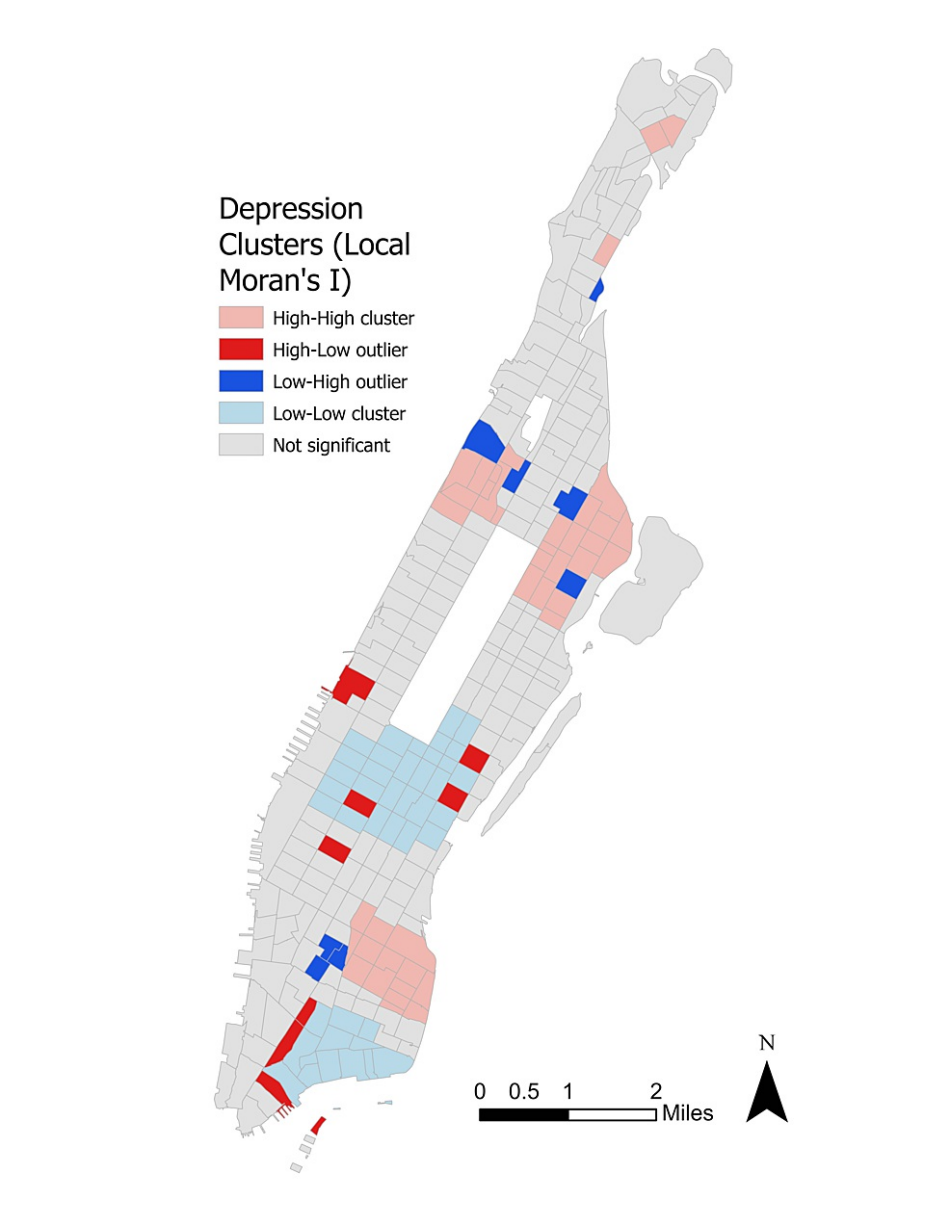
Local Moran's I spatial autocorrelation results.

To further investigate the spatial distribution of depression incidence, spatial autocorrelation was used to identify outliers. Figure 5 displays the results of the Local Moran’s I spatial autocorrelation analysis.

The Local Moran’s I analysis does buttress much of what was seen in the Getis-Ord Gi* map in Figure 4. However, some noticeable outliers are seen among these clusters. Low depression rates clustered among depression hot spots in northeastern Washington Heights, northern Morningside Heights, western and southern East Harlem, and Greenwich Village just west of Gramercy. High depression rates clustered around depression cold spots in western Lincoln Square, eastern and southern Midtown, and western Wall Street. However, Local Moran’s I outlier identification did not visually identify any large-scale spatial relationship between race and depression, though it did confirm the observed relationships in Upper Manhattan seen in the Getis-Ord Gi* analysis.

Geographically weighted regression

While spatial clustering analysis is useful in identifying distribution trends in depression rates, it does not provide predictive power for why depression rates spatially concentrate in certain areas and which factors may affect said concentrations. To analyze this, a GWR was estimated using the eight predictive variables discussed in the methodology. Before the GWR could be modeled, statistically significant parameters at $\alpha = 0.05$ were identified via a GLR using ordinary least squares (OLS). Below is the multivariate cross-sectional OLS model used to fit the GWR, where $D_{2020}$ is the depression rate, $x_{1}...x_{8}$ are the exogenous parameters, $\tau _{0}$ is the equation constant, $\tau _{1}...\tau_{8}$ are the coefficient magnitudes for the predictive variables, and $\varepsilon_{0}$ is the error term, or the variance of the model from the predicted depression values:

$D_{2020}=\tau_{0}+\tau_{1}x_{1}+\tau_{2}x_{2}+...+\tau_{8}x_{8}+\varepsilon _{0}$

The following initial results were returned from the model as seen in Table 2. Robust standard errors and p-values were reported to control heteroskedasticity.

**Table 2:** Initial generalized linear regression results. Parameters: Insurance: lack of health insurance, drinking: binge drinking, checkups: routine doctor visits, smoking: those reporting regular cigarette use, inactivity: those physically inactive, distress: frequent mental distress, obesity: those with a body mass index (BMI) of 30.0 kg/m^2^ or greater, deprivation: sleep deprivation. P-value: Probability value. R^2^: Coefficient of determination.

Adjusted R^2^	0.9357			
Observations	281			
Parameter	Coefficient	Robust error	Robust P-value	Variance inflation factor (VIF)
Insurance	-0.18	0.04	0.001	72.28
Drinking	0.06	0.08	0.463	37.33
Checkups	-0.01	0.07	0.915	24.04
Smoking	0.10	0.08	0.208	31.44
Inactivity	0.04	0.05	0.470	133.96
Distress	1.06	0.05	0.001	10.14
Obesity	0.25	0.05	0.001	37.19
Deprivation	-0.55	0.02	0.001	15.18
Intercept	17.27	6.27	0.006	n/a

For the variance inflation factor (VIF), which measures collinearity between the exogenous parameters, values > 5 are considered collinear. Ergo, all variables insignificant at $\alpha =0.05$ or with a VIF > 5 were removed in a stepwise manner from the final OLS estimation. Table 3 displays the final results and their corresponding summary values.

**Table 3:** Final generalized linear regression results. Parameters: Insurance: lack of health insurance, distress: frequent mental distress. P-value: Probability value. R^2^: Coefficient of determination.

Adjusted R^2^	0.5639			
Observations	281			
Parameter	Coefficient	Robust error	Robust P-value	Variance inflation factor (VIF)
Insurance	-0.12	0.02	0.001	3.39
Distress	0.73	0.05	0.001	3.39
Intercept	10.93	0.56	0.001	n/a

**Figure 6:**
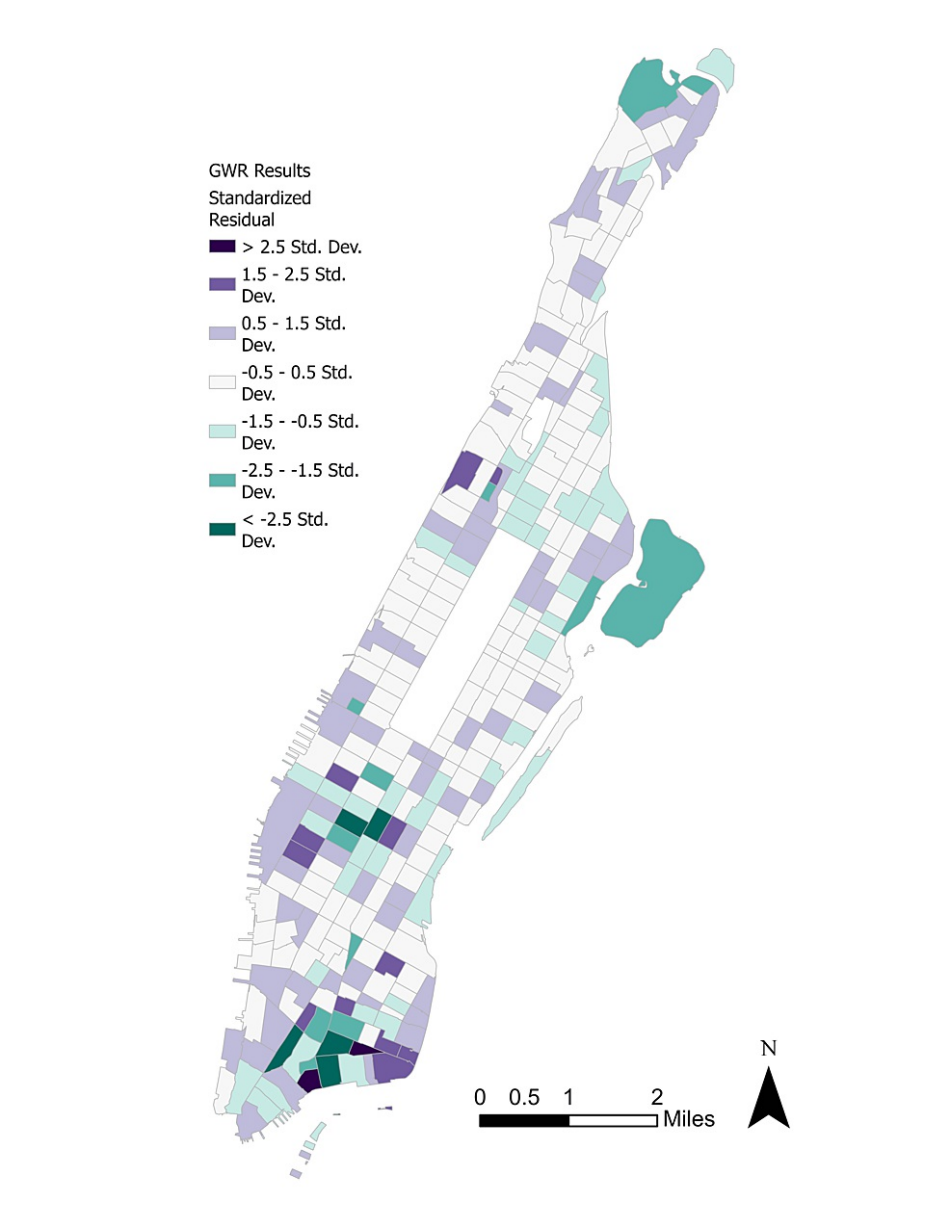
Regression standardized residuals. GWR: Geographically weighted regression, Std. Dev.: Standard deviation.

**Figure 7:**
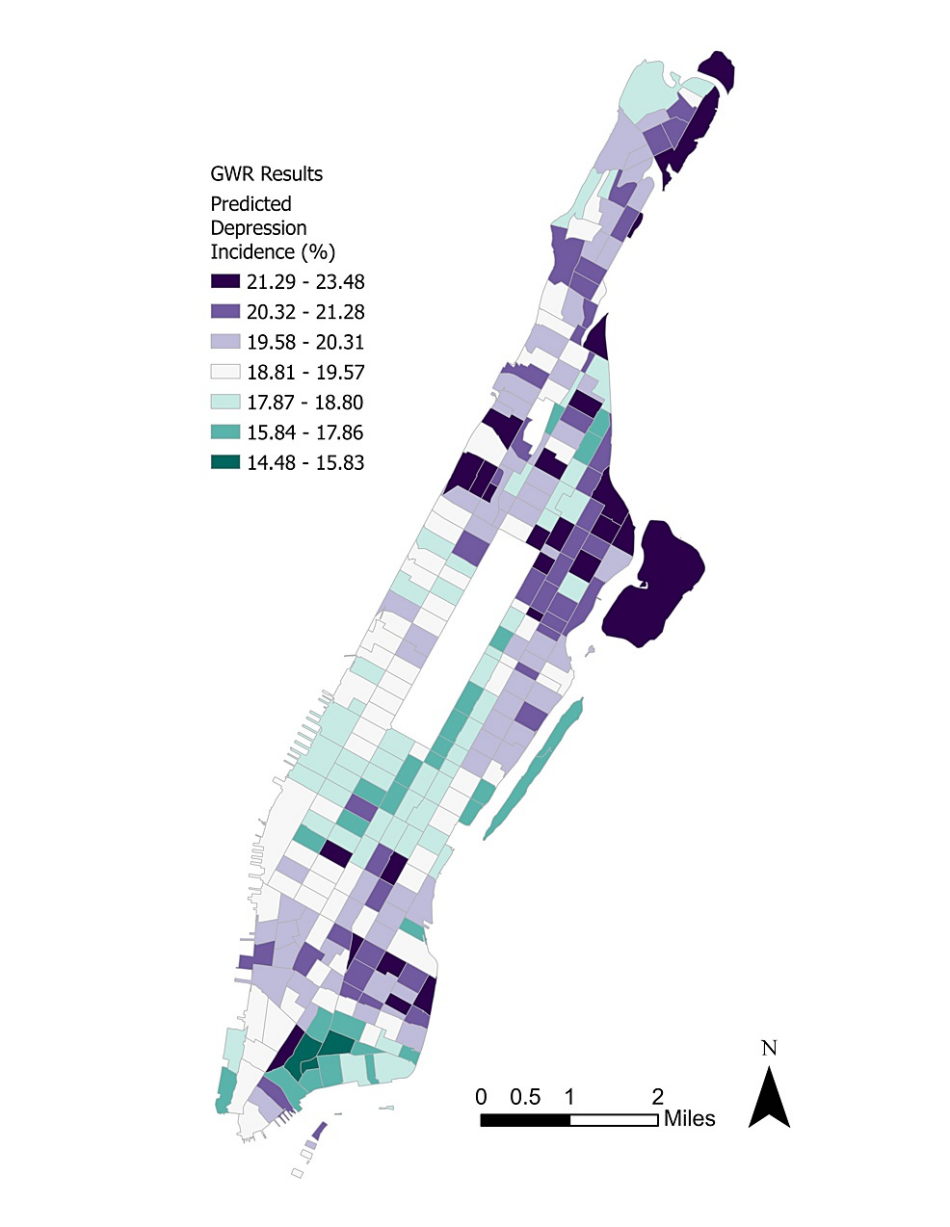
Regression-predicted depressive disorder incidence. GWR: Geographically weighted regression.

Since only the lack of insurance and frequent mental distress parameters were significant at $\alpha =0.05$, the GWR was fitted using these variables. Figure 6 displays the spatial distribution of the GWR standardized residuals and Figure 7 shows the predicted depression incidence distribution. 

**Figure 8:**
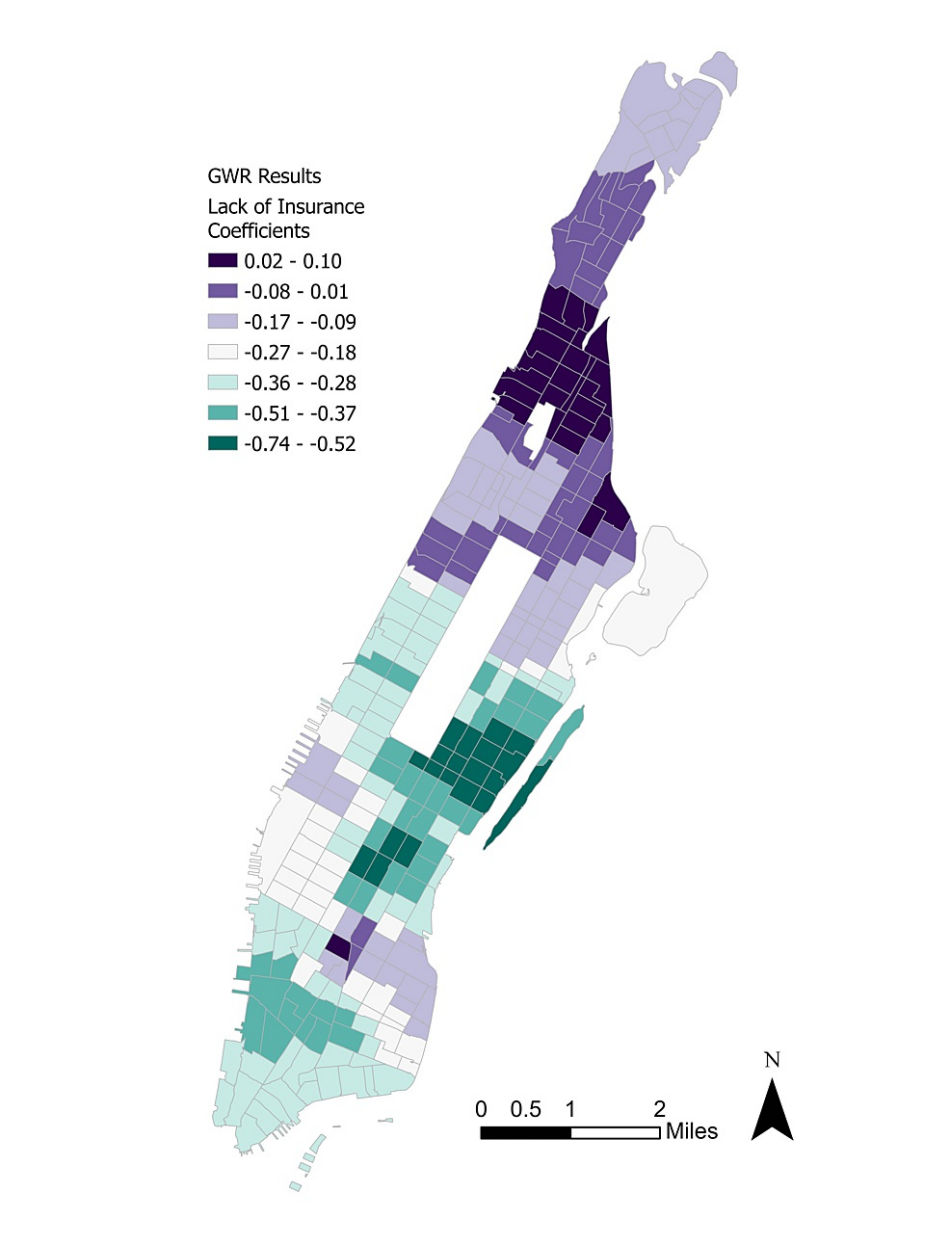
Distribution of lack of insurance coefficients. GWR: Geographically weighted regression.

**Figure 9:**
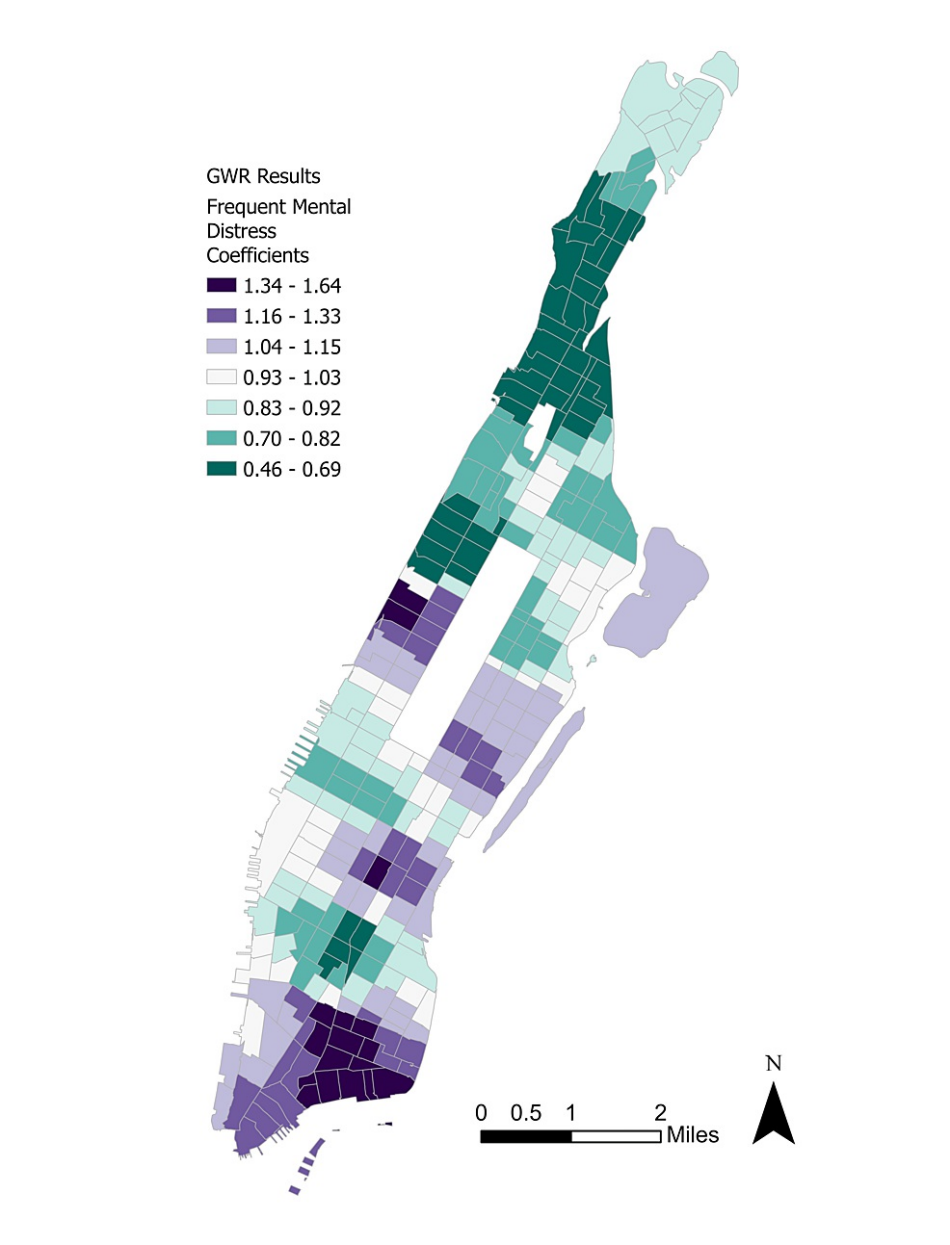
Distribution of frequent mental distress coefficients. GWR: Geographically weighted regression.

There are extensive differences between the observed and predicted depression rates by tract, with higher observed rates in Upper Manhattan and parts of Gramercy. However, the observed depression rates were typically 1-2 standard deviations below the predicted rates in the Lower Manhattan neighborhood of Wall Street. Coefficient maps for the two exogenous parameters are displayed in Figure 8 and Figure 9.

A rough inversion of the coefficient magnitudes is visible, with a more spatial concentration of positive insurance effects on depression in Upper Manhattan’s Washington Heights and a more spatial concentration of positive distress effects on depression in Lower Manhattan’s Wall Street. These distributions help to explain the seemingly random dispersion of depression incidence across Manhattan.

## Discussion

The use of Getis-Ord Gi* allowed for the initial identification of hot spots and cold spots of depression while Local Moran’s I helped isolate any high or low depression outlier neighbors. These spatial distribution analyses helped determine important geographic trends that would otherwise have been undetectable when viewing the simple graduated choropleth of depression incidence in Figure 3. The extreme clustering of high depression incidence in either majority non-White neighborhoods or neighborhoods with substantial non-White populations is critical from a public health standpoint. As non-White New Yorkers in Upper Manhattan continue to face many hurdles to accessing mental health treatment, diverting capital planning resources to these acute urban blocks may reduce the level of clinical depression in these areas.

The use of Local Moran’s I in identifying outliers proved vital for two reasons. First, it may allow for better resource allocation to overlooked neighborhoods. For example, the high depression incidence in eastern SoHo and Tribeca, and the northern Financial District are directly adjacent to the cold spots throughout Wall Street. Therefore, these neighborhoods, which may be ignored in the general allocation of health resources, will now be identifiable to city officials in the future. Second, it also reveals the extreme level of mental health segregation and disjoint in dense urban regions. The sheer number of outliers directly adjacent to major clusters in such dense urban blocks reveals that despite the overall positive mental health benefits bestowed on residents by urbanization, certain populations may be left out of these positive externalities.

A GWR helped to identify not only spatial parameters for depression incidence but predicted incidence based on statistically significant parameters. GWR also permitted examination of the spatial patterns of individual exogenous parameters and their magnitudes, as well as the magnitudes of the standardized residuals for depression incidence. Interestingly, Upper Manhattan’s GWR-estimated rates were on average 0.5-1.0 standard deviations lower than the observed values. Although the standardized residuals were not substantially large with a mean of -0.01 and median of 0.05, the results show that statistically significant parameters alone cannot explain depressive disorder rates; urbanization forces such as segregation and gentrification may play outsized roles.

Finally, the rough inversion in the lack of insurance and mental distress coefficients is intriguing. One possible explanation is that majority non-White neighborhoods in Upper Manhattan such as Harlem and Washington Heights tend to also be low-income and therefore have lower rates of health insurance enrollment based on the PLACES datasets. Majority White neighborhoods generally tend to be more affluent and have greater health insurance access [20]. Therefore, it was predicted that the lack of insurance would correlate positively with depression in neighborhoods with high uninsured rates while it would have a negative or minuscule effect in more affluent neighborhoods. On the other hand, majority White neighborhoods may be more likely to self-report frequent mental distress whereas majority non-White neighborhoods have historically been undercounted in government surveys like the U.S. census, which leads to a skewed distribution of federal resources [21]. Therefore, the effect of distress on depression would be more acutely felt in majority White neighborhoods in Lower Manhattan than in majority non-White neighborhoods in Upper Manhattan. The main exception to this conclusion is Manhattan Valley, which has an overwhelmingly non-White residential population and a relatively higher depression rate. However, this neighborhood is experiencing rapid gentrification and a growing White population, which may partially explain its outlier status [22]. 

Limitations

There were several limitations identified in this study. For one, none of the models made full use of the demographic data discussed. The intent was to focus exclusively on healthcare and economic characteristics, or parameters that carry direct public resource allocation implications. Nonetheless, analyzing the exogenous impacts of race and ethnicity on depression rates may have improved the spatial analyses carried out on depression incidence. A second limitation is the number of outliers identified. While the presence of many outliers in and around clusters supports the impact of segregation on depression incidence, the sheer level of disjoint seen in the analysis makes direct policy answers cumbersome and requires a tract-by-tract analysis of resource allocation.

Another limitation is the applicability of the results. While the analysis is broadly applicable to dense urban cores similar to Manhattan, it may fall short in relation to medium-sized cities and localities. Nevertheless, as many urban regions find themselves trending towards a similar population density as Manhattan, this study may prove useful in the analysis of future urban health trends. 

There are several avenues for future research. For one, a more robust investigation of the impact of gentrification on healthcare access in urban centers may complement this study. While gentrification may displace low-income residents, it may also bring new community facilities such as healthcare providers. Another avenue for research is to investigate the underreporting that often complicates government datasets such as the census and PLACES, which may determine the provision of public finance and lead to healthcare "deserts." A potential intersection analysis of healthcare facilities in Manhattan with the results of this study may help to identify areas of high healthcare need. 

## Conclusions

Overall depression incidence varies by neighborhood across the island of Manhattan, with majority White, more affluent neighborhoods in Lower and Midtown Manhattan experiencing less overall depression than majority non-White, less affluent areas to the north. However, the gentrification and segregation seen across Manhattan have led to many noticeable outlier blocks near these large clusters. While reversing the negative spillovers associated with these two forces will require long-run capital planning, city health officials can begin by targeting mental health treatment resources in majority non-White areas with high uninsured rates and those areas with frequent mental distress irrespective of racial or ethnic neighborhood character.
